# YTHDF2 regulates ACSL4-dependent ferroptosis of keratinocytes in diabetic wound healing

**DOI:** 10.1042/CS20255877

**Published:** 2025-08-20

**Authors:** Liangyan Wu, Lanlan Li, Wei Wang, Sifan Chen, Phei Er Saw, Xiaosi Hong, Diefei Liang, Chen Yang, Li Yan, Wei-Jye Lin, Meng Ren

**Affiliations:** 1 Department of Endocrinology, Sun Yat-Sen Memorial Hospital, Sun Yat-Sen University, Guangzhou, 510120, China; 2 Department of Endocrinology and Metabolism, The First Affiliated Hospital of Jinan University, Guangzhou, 510630, China; 3 Guangdong Provincial Key Laboratory of Malignant Tumor Epigenetics and Gene Regulation, Guangdong-Hong Kong Joint Laboratory for RNA Medicine, Medical Research Center, Sun Yat-Sen Memorial Hospital, Sun Yat-Sen University, Guangzhou, 510120, China; 4 Nanhai Translational Innovation Center of Precision Immunology, Sun Yat-sen Memorial Hospital, Foshan, 528200, China; 5 Department of Endocrinology and Metabolism, Zhuhai People’s Hospital (Zhuhai Hospital Affiliated with Jinan University), Zhuhai, 519000, China; 6 Brain research center, Sun Yat-Sen Memorial Hospital, Sun Yat-Sen University, Guangzhou, 510120, China; 7 Guangdong Province Key Laboratory of Brain Function and Disease, Zhongshan School of Medicine, Sun Yat-Sen University, Guangzhou, 510120, China; 8 Medical Research Center, Sun Yat-Sen Memorial Hospital, Sun Yat-Sen University, Guangzhou, 510120, China

**Keywords:** ACSL4, ferroptosis, diabetic wound healing, YTHDF2

## Abstract

Delayed diabetic wound healing is a global health issue with unclear pathogenesis. Ferroptosis, a form of cell death involving iron and lipid peroxidation, may contribute to delayed diabetic wound. This study investigates the role of ferroptosis in diabetic wound keratinocytes. We measured lipid peroxidation products (MDA, 4-HNE), ACSL4, and GPX4 protein levels in diabetic keratinocytes and assessed mitochondrial morphology. Ferrostatin-1 (Fer-1) was used to inhibit ferroptosis in diabetic rat wounds, and its effects on healing and expression levels were evaluated. Pull-down assays, silver staining, and mass spectrometry were employed to study ACSL4 mRNA regulation. A YTHDF2 knockdown adenovirus was used to manipulate YTHDF2 expression in rat wounds. Ferroptosis was detected in diabetic keratinocytes, hindering wound healing, a process reversible with Fer-1. High glucose induced ACSL4 expression, driving keratinocyte ferroptosis and delayed healing. YTHDF2 interacts with N6-methyladenosine-modified ACSL4 mRNA, affecting its stability and expression. YTHDF2 knockdown increased ACSL4, promoting ferroptosis and impairing healing. Our findings illustrate the significant involvement of ferroptosis in the dysfunction of diabetic keratinocytes, suggesting that targeting ferroptosis may offer a viable therapeutic approach for improving diabetic wound healing.

## Introduction

The rates of occurrence, frequency, and fatality of diabetes are on the rise, presenting a major global health hazard [1]. Approximately 25% of individuals suffering from diabetes mellitus are affected by the delayed closure of diabetic wounds [2,3]. Moreover, delayed diabetic wound healing is the primary cause of amputation in patients with diabetes, which decreases the 5-year survival rate to approximately 43% [4] and becomes a serious economic burden on society [5]. The therapeutic effects of existing approaches, including surgical debridement, graft transplantation, wound dressing, physical hyperbaric oxygen therapy, and so on [6], remain unsatisfactory. Therefore, a better understanding of the mechanisms underlying impaired wound healing in diabetes will facilitate the development of more effective therapeutic approaches.

The epidermis of the skin of patients with diabetes is notably thinner and more prone to injury than that of controls [7], leading to delayed wound healing. Keratinocytes, the main epidermis components, may decrease under diabetic conditions. Keratinocytes are involved in the formation of a protective barrier through proliferation, differentiation, and migration [8] and play a key role in the wound healing process. Moreover, several studies have reported that diabetic wound healing can be rescued by improving the function of keratinocytes in diabetic conditions [9]. Existing research indicates that elevated blood glucose levels induce oxidative stress by enhancing the production of reactive oxygen species (ROS), thereby compromising the normal functions of keratinocytes and potentially leading to cell death [8,10,11] and ultimately leading to delayed diabetic wound healing. Moreover, the expression of malondialdehyde (MDA), a lipid peroxidation product, is elevated in diabetic keratinocytes [12]. Ferroptosis, a newly discovered type of cell death that relies on iron, is marked by the process of lipid peroxidation [13]. Ferroptosis fundamentally manifests through iron-driven phospholipid oxidation and disrupted redox homeostasis, collectively provoking membrane damage and cell death. Studies have reported that the cysteine/glutathione/glutathione peroxidase 4 (GPX4) axis [14], iron metabolism, and lipid metabolism as a tripartite regulatory framework for the concerted modulation of this cell death [15,16]. Several investigations have explored the connection between ferroptosis and diabetic wounds, albeit limited in number [17], the detailed pathogenic mechanisms underlying ferroptosis in diabetic keratinocytes remain elusive.

ACSL4, an enzyme responsible for facilitating the transformation of fatty acids into fatty acyl-CoA esters, has been identified as a specific ferroptosis biomarker and driver because ACSL4 up-regulation enhances the polyunsaturated fatty acid content in phospholipids, which are susceptible to oxidation reactions that trigger ferroptosis [18]. The ability of ACSL4 to induce ferroptosis in diabetes-related complications has been reported. Research conducted earlier indicates that the expression of ACSL4 is elevated in the context of diabetic nephropathy and diabetic cardiomyopathy, leading to ferroptosis [19–21]. Considering these findings, it is reasonable to speculate that ferroptosis may occur in the keratinocytes of diabetic skin. Whether this mechanism is associated with ACSL4-mediated ferroptosis requires further investigation.

The mechanistic basis for altered ACSL4 expression, especially mRNA, under high-glucose conditions remains unverified. The N6-methyladenosine modification has been identified as one of the most important post-transcriptional regulatory types of RNAs. N6-methyladenosine modulates virtually all mRNA metabolic processes, including spanning splicing mechanisms, transcript stability, translational efficiency, and microRNA biogenesis [22–24]. Several studies have reported the multifunctional roles of N6-methyladenosine in encompassing cellular differentiation, immunological equilibrium, mitotic regulation, metabolic disorders, oncogenesis, and circadian rhythm maintenance [25–27]. This reversible epigenetic mark is dynamically regulated by a tripartite protein system: methyltransferases (‘writers’), demethylases (‘erasers’), and recognition proteins (‘readers’) [28,29]. Writer complexes catalyze N6-methyladenosine installation while eraser enzymes mediate its removal, establishing bidirectional regulatory control. Existing studies on the role of N6-methyladenosine modification in diabetic wound healing have predominantly focused on its regulation of cellular functions through autophagy [30,31]. Whether N6-methyladenosine modification contributes to diabetic wound healing by mediating ferroptosis remains rigorously validated. A previous study has shown that N6-methyladenosine levels are lower in diabetic keratinocytes than in controls [32]. YTHDF2, an N6-methyladenosine reader, recognizes N6-methyladenosine-containing mRNAs to regulate their stability [33]. Although our previous study found that YTHDF2 can increase the translational activity of the mRNA of matrix metalloproteinase-9 and induce collagen degradation for wound healing [34], it remains unknown whether this reader can regulate N6-methyladenosine-modified ACSL4 mRNA degradation and ultimately promote ferroptosis in the keratinocytes of diabetic skin.

We examined the phenotypes of ferroptosis in diabetic tissues and high-glucose-stimulated keratinocytes and investigated associated signaling pathways to elucidate the therapeutic potential of targeting ferroptosis for modulating keratinocyte functionality. This investigation revealed an increase in ACSL4 expression within diabetic skin, thereby causing ferroptosis in keratinocytes. Moreover, we revealed that the N6-methyladenosine reader YTHDF2 regulates ferroptosis by binding to ACSL4 mRNA and mediating ACSL4 expression. These findings present significant implications regarding ferroptosis for both fundamental research progress and clinical translation in addressing diabetic wound healing.

## Materials and methods

### Human tissue samples

Skin specimens adjacent to lesions (*n* = 3) were harvested from patients with diabetes mellitus requiring amputation. In contrast, normal skin samples (*n* = 3) were procured from the lower limbs of individuals without diabetes who were undergoing corrective surgery for foot trauma. Then, 1 cm skin biopsy punches were employed to collect skin tissues. Additionally, exudate samples from diabetic foot ulcers (*n* = 6) were collected at the ulceration sites in patients with diabetic foot ulcers. Similarly, exudate from nondiabetic individuals (*n* = 6) was gathered from those suffering from chronic venous ulcers in the lower extremities, who did not have diabetes. All research was carried out in accordance with the guidelines of the Declaration of Helsinki II, following a protocol that was authorized by the Institutional Review Board at Sun Yat-sen Memorial Hospital, which is affiliated with Sun Yat-sen University (SYSKY-2023–279-01). All participants signed a written consent form after being fully informed.

### Animals

Male Sprague-Dawley (SD) rats of the wildtype (WT), with a body weight ranging from 140 to 160 g, were acquired from the Laboratory Animal Center of Sun Yat-sen University. The rats were reared and cared for in the Laboratory Animal Center at Sun Yat-sen University, where they were kept in a pathogen-free environment. They were provided with standard rations of food and water, and the facility adhered to a 12 hour light-to-dark cycle regimen. The diabetic model was induced through an intraperitoneal administration of streptozotocin (STZ, Sigma, U.S.A.) at a dose of 60 mg/kg. After 72 h, the rats achieved the standard of diabetes when the glucose concentrations were greater or equal to 16.7 mM. A diabetic skin wound (1 or 2 cm) was made on the dorsal skin of rats, as previously described [35]. Following the conclusion of the experiment, the mice were killed using halothane anesthesia, and 1 cm skin biopsy punches were employed to collect wound tissues. Subsequent to this, their skin samples were collected for additional analysis. All animal experiments were conducted in accordance with the ARRIVE guidelines. The Institutional Animal Care and Use Committee of Sun Yat-sen University granted approval for all animal studies conducted (approval number: SYSU-IACUC-2023–000319).

#### *In vivo* evaluation of ferroptosis level

To assess the ferroptosis status in both diabetic and normal skin, rats with and without diabetes were allocated into two separate groups, each consisting of four animals. Following euthanasia, the skin from these rats was harvested for subsequent ferroptosis analysis.

To investigate the function of inhibitors targeting ferroptosis in the process of diabetic wound recovery, normal control and STZ-induced diabetic rats with 1 cm skin wounds were divided into three groups (*n* = 4 rats/group): untreated WT rats, untreated diabetic rats, and ferroptosis inhibitor ferrostatin-1 (Fer-1)-treated diabetic rats. Diabetic rats were injected intradermally with Fer-1 (#S7243, Selleck, U.S.A.) (15 μmol/l, 100 μl/rat), and the corresponding negative control solvent once every other day for 10 consecutive days, and control rats also received the corresponding negative control solvent. The wounds were photographed on days 0, 2, 4, 6, and 8 after the solvent injection. The wounds were quantified by the Image J software.

#### *In vivo* wound healing model

To study the effects of YTHDF2 reduction *in vivo*, WT Sprague-Dawley rats were allocated into two groups at random (*n* = 5). A 2 cm full-thickness skin wound was made on the backs of the rats using a biopsy punch. The 5 × 10^9^ pfu YTHDF2-knockdown YTHDF2-knockdown Adenovirus was injected intradermally around the wound circle 0 day after the wound was made. On days 0, 3, 6, 9, and 12 after the wound was made, photos of the wound models were taken and quantified using the ImageJ software. Wound healing progression was quantified using the baseline wound dimension (day 0 measurement) as reference, with percentage closure mathematically expressed as: (wound area on day X/ original wound area) × 100%.

### MDA detection

The level of MDA in tissues was measured to reflect the extent of lipid peroxidation. The MDA content was quantified with the aid of TBARS Assay kit (#700870–96, Cayman Chemical, U.S.A.) per the manufacturer’s instructions.

For MDA detection in the exudate, sterile gauze was plated on the wound after cleaning the diabetic foot wound using saline and drying. The exudate-wetted sterile gauze was then centrifuged in a microcentrifuge tube to obtain the exudate. The MDA content in the exudate was assessed by the TBARS Assay kit.

Fresh skin tissues were obtained from control and diabetic rats to determine MDA levels in the skin. Then skins were incubated in dispase II (#4942078001, Roche, Swit) overnight at 4℃ to separate the epidermis and dermis. The separated epidermis was incubated in RIPA buffer (#CW2333, CWBIO, CHINA) with a protease and phosphatase inhibitor cocktail (#CW2200S, CWBIO, China) to extract total protein. Subsequently, the protein supernatants were harvested, and the MDA levels were measured with the TBARS Assay kit.

### Cell line

The Human keratinocyte cell line, HaCaT, was cultured in Minimum Essential Medium (MEM) (#PM150410, Procell, China) with 10% Fetal bovine serum (FBS) (#FSP500, ExCell Bio, U.S.A.) and cultured at the same condition.

### Primary keratinocyte isolation

Human skin specimens were procured from individuals who had previously undergone surgical removal of the foreskin. The skins were collected and incubated in dispase II overnight at 4℃ to separate the epidermis and dermis. Then, the epidermis was trypsinized at 37℃ for 30 minutes, and the digestion was terminated with a Trypsin Neutralization Solution (TNS) (#0311, ScienCell, U.S.A.). The cell pellet was obtained by centrifugation at 1000×g. Subsequently, the cells were re-suspended and grown in a medium specifically designed for keratinocytes (KM) (#2101; Scientific Cell, USA). All cells were cultured in a 37℃, 5% CO_2_ incubator. For experiments, primary keratinocytes and HaCaT cells received glucose (G6152, Sigma, U.S.A.) at defined concentrations: normal (5.6 mM), mid-high (16.5 mM), and high (33 mM). Mannitol (M4125, Sigma, U.S.A.) (16.5 mM: 5.6 mM of glucose + 10.9 mM of mannitol; 33 mM: 5.6 mM of glucose + 27.4 mM of mannitol）was employed as an osmotic control.

### Plasmid transfection

WZ Biosciences produced vectors for ACSL4 overexpression and control. HaCaT cells were then seeded in 12-well plates. Following the protocol provided by the manufacturer, 1 μg of plasmid DNA was introduced into the HaCaT cells with the aid of Lipofectamine 3000 transfection reagent (#L3000015, ThermoFisher, U.S.A.).

### RNA interference

RiboBio crafted and produced the small interfering RNAs (siRNAs). Subsequently, the cells were plated into a 12-well dish. In accordance with the manufacturer’s guidelines, 100 pmol of siRNA was delivered into the cells using Lipofectamine RNAiMAX transfection reagent (#13778150, Invitrogen, USA). Supplementary Table S1 presents the sequences of siRNAs.

### Cell viability assay

The Cell Counting Kit-8 (CCK8, product number K1018, APE×BIO, USA) was employed to evaluate cellular viability. A total of 10^4^ cells were seeded into 96-well plates, followed by the addition of 10 μl of CCK-8 reagent combined with the culture medium to each well. Subsequently, the plates were incubated at 37℃ for a period of 2 hours. The absorbance at 450 nm was then recorded using a microplate reader.

### Intracellular ferrous ions analysis

Intracellular levels of ferrous ions in HaCaT were detected using FerroOrange probes (#F374, Dojindo, Japan). HaCaT cells were cultured in 12-well plates and subjected to the required treatments. Prior to the assay, the cells were rinsed thrice with a serum-free medium. They were then incubated for one hour at 37°C with a 1 μM working solution of FerroOrange probes, diluted in serum-free medium. Subsequent to the incubation, the cells were examined under a fluorescence microscope without any further washing.

### Flow cytometric analysis

To ascertain cell death, the cells were harvested, rinsed using PBS (#SH30256.01B, Hyclone, AUS), re-suspended in a buffer for binding, and then labeled with annexin V conjugated to FITC and propidium iodide (#E-CK-A211-50T, Elabscience, China). Finally, flow cytometry (Beckman Coulter, Indianapolis, IN, U.S.A.) was used to process the cells, and the results were analyzed.

### BODIPY™ 581/591 C11 assay

The BODIPY 581/591 C11 dye (#D3861, Thermo Fisher Scientific) was utilized to assess lipid peroxidation levels. The cells were treated with 1 μM/mL of BODIPY™ 581/591 C11 and incubated at 37°C for a duration of 40 minutes, shielded from light as recommended by the product instructions. Following incubation, the cells were subjected to two washes with PBS. Fluorescence intensity was quantified using a flow cytometer with excitation/emission settings at 488/565 nm. Oxidation of the dye resulted in a shift of the emission maximum to 510 nm from the original 590 nm.

### RNA isolation, quantitative real-time PCR analysis

RNA was extracted using the TRIzol reagent (#9109; Takara, Japan) as directed by the supplier. cDNA was generated from 500 ng of the extracted RNA with a reverse transcriptase kit (#AG11706; AGBIO, CHINA). Quantitative real-time polymerase chain reaction (RT-qPCR) (#AG11701, AGBIO, CHINA) was carried out on a Roche LightCycler 480 instrument with SYBR Green I Master Mix. The mRNA expression levels were normalized to β-actin, and the data were analyzed with the 2^-△△ct^ method. Supplementary Table S2 presents the sequences of primers used.

### Western blot analysis

The cells and skin tissues were lysed using RIPA buffer supplemented with a mixture of protease and phosphatase inhibitors. For protein sample preparation, SDS-Blue loading buffer was added, followed by incubation at 95℃ for a duration of 10 minutes. Proteins were separated using sodium dodecyl sulfate-polyacrylamide gel electrophoresis and transferred onto polyvinylidene fluoride membranes according to standard protocols. The membranes were first blocked with 5% nonfat dry milk for 1 hour at ambient temperature before being subjected to incubation with the anti-β-actin (1:1000, #GB11001, Servicebio, China), anti-YTHDF2 (1:5000, #24744, Proteintech, China), and anti-ACSL4 (1:10000, #ab155282, Abcam, UK) antibody dilutions at 4℃ overnight. After being washed with Tris-buffered saline, the membranes were subjected to a one-hour incubation at room temperature with either horseradish peroxidase-labeled anti-mouse (1:1000, #A0216, Beyotime, China) or anti-rabbit secondary antibodies (1:1000, #A0208, Beyotime, China). Subsequently, the protein bands were visualized by applying the Immobilon Western HRP Substrate (#WBKLS0500, Millipore, Germany).

### RNA stability assay

For assessing RNA stability, Actinomycin D (#A4448, APExBIO, U.S.A.) was utilized. The HaCaT cells were placed in 12-well dishes. Following the appropriate treatments, Actinomycin D was introduced at a concentration of 5 μg/ml to inhibit mRNA synthesis. At 0, 3, 6, and 9 hours post-treatment, the cells were harvested for the analysis of target gene mRNA levels and to assess their stability via RT-qPCR.

### Pull-down assay

According to the pull-down assay, the ACSL4 probe was used to pull proteins interacting with it. RiboBio was responsible for the design and synthesis of biotinylated probes, both random and specific to ACSL4 RNA. For pull-down assays, 10^7^ HaCaT cells were collected. Biotinylated random and ACSL4 RNA probes were added to the pretreated streptavidin magnetic beads (#HY-K0202, MedChemExpress, U.S.A.). After 1 hour of co-incubation at room temperature, the probe-coated beads complex was added with protein lysate to prepare an RNA–protein complex at 4℃ overnight. The next day, 50 μl Elution Buffer was used to elute the RNA–protein complex from beads at 37℃ for 30 minutes. Eluted proteins were subjected to western blotting and mass spectrometry. The related raw data of mass spectrometry have been deposited to the protein database PRIDE [36]. Supplementary Table S3 presents the sequences of used biotinylated probes.

### RNA immunoprecipitation assay

The antibodies were used in immunoprecipitation assay to capture its interacting RNAs. A total of 10^7^ HaCaT cells were subjected to a double washing with PBS, subsequently harvested in PBS, and then lysed in RIPA buffer. Following this, 100 μl of the lysate was mixed with rip buffer that included magnetic beads for incubation (#HY-K0202, MedChemExpress, U.S.A.) conjugated to 5 μg anti-N6-methyladenosine (#ab151230, Abcam, UK) and 5 μg anti-YTHDF2 antibodies. Purified anti-rabbit IgG antibody (#A7016; Beyotime, China) was used as a negative control. RNAs immunoprecipitated were then extracted and purified in preparation for RT-qPCR to quantify ACSL4 expression levels.

### Wound-healing assay

HaCaT cells were plated into 6-well dishes and then transfected with either ACSL4 or control plasmid constructs. After 72 hours, a scratch was created in the monolayer of adherent cells using a 200 μl pipette tip. HaCaT cells were imaged 0 hour after the scratch operation was completed. The cells were further cultured for 16 and 24 hours, and the same scratch was captured. The migration width was measured by the ImageJ software.

### ROS detection

The levels of ROS in skin tissues, especially in epidermis, were assessed using Dihydroethidium (DHE) (#S0063, Beyotime, China). Tissue sections were deparaffinized and hydrated using xylene and ethanol. Next, DHE was added to the sections at a dilution of 1:1000 and incubated at 37℃ for a duration of 40 minutes in the dark. Subsequently, the tissue sections were subjected to three rinses with PBS. Nuclei were then counterstained using DAPI (#B0011; Baiqiandubio, China) for 10 minutes. After three washes with PBS, an anti-fluorescence quenching agent (#B0008; Baiqiandubio, China) was used for sealing. Images of fluorescence were obtained through a fluorescent microscope.

### Immunohistochemical staining

Tissue sections on slides were heated at 68℃ for 2 hours, followed by deparaffinization and rehydration through a series of xylene and ethanol treatments. The slides were then placed in EDTA solution and heated to 95°C for a 20 minute antigen retrieval process. Afterward, a blocking solution was applied to the tissue sections for 30 minutes at room temperature to prevent non-specific binding. Subsequently, the slides were incubated overnight at 4℃ with primary antibodies against YTHDF2 (1:250), ACSL4 (1:250), and 4-Hydroxynonenal (4HNE) (1:150, #MAB3249, R&D systems, U.S.A.). The next day, the slides were treated with horseradish peroxidase (HRP)-conjugated secondary antibodies, either anti-mouse (1:100) or anti-rabbit (1:100), for 1 hour at room temperature. This was followed by incubation with 3,3-diaminobenzidine (DAB) (#DA1010, Solarbio, China) and hematoxylin for staining. The positive staining areas were quantified using ImageJ analysis software.

### Hematoxylin–eosin staining and Masson’s trichrome staining

Skin samples were preserved in 4% paraformaldehyde for a duration of 24 hours, subsequently dehydrated, and then embedded within paraffin wax. Subsequently, sections of 4 μm in thickness were cut from the paraffin blocks for hematoxylin–eosin (HE) and Masson’s trichrome (MTC) staining procedures. Measurements of epidermal depth, wound breadth, and the area of MTC-positive skin were conducted utilizing Image J analysis software.

### Statistical analysis

Data analysis was conducted using GraphPad Prism, and the results are presented as mean ± standard deviation (SD) or mean ± standard error of the mean. To assess the statistical significance of the differences observed, Student’s *t*-test and one-way analysis of variance were applied, with subsequent use of the least significant difference *t*-test or Dunnett’s test for post-hoc comparisons. A *P* value <0.05 was considered statistically significant.

## Results

### Ferroptosis occurred in diabetic skin

**Figure 1:**
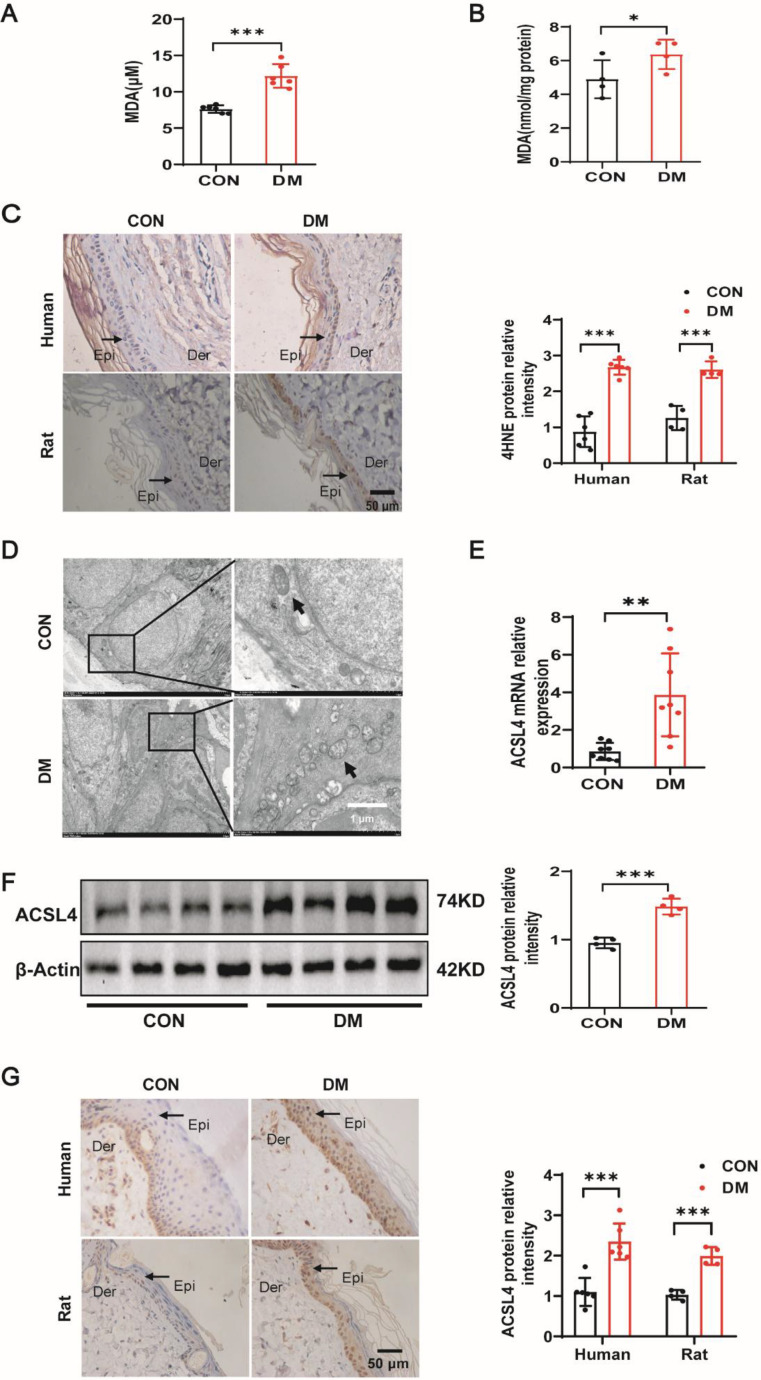
Ferroptosis occurred in diabetic skin. (**A**) MDA contents were detected in the wound exudates from control and patients with diabetes mellitus (*n* = 6). (**B**) MDA contents were detected in the skin tissues of control and diabetic rats (*n* = 4). (**C**) Histological analysis to detect 4HNE levels in skin tissues from patients with diabetes mellitus (*n* = 6) and diabetic rats (*n* = 4). Original magnification × 400. (**D**)TEM analysis of skin tissues from diabetic rats and control. Arrowheads indicate mitochondria. (**E and F**) The skin tissues from diabetic rats and controls were subjected to qPCR of ACSL4 (**E**) and immunoblotting (**F**) (*n* = 4). (**G**) Skin tissues from patients with diabetes mellitus (*n* = 3) and diabetic rats (*n* = 4) were subjected to Histological analysis of ACSL4. Original magnification × 400. Bars represent the mean ± SD; *P < 0.05; **P < 0.01; ***P < 0.001. ACSL4, acyl-CoA synthetase long-chain family member 4; MDA, malondialdehyde.

HE staining revealed that the skin of diabetic individuals and rats with STZ-induced diabetes exhibited considerable reduction in thickness (Supplementary Figure S1A). To confirm the involvement of ferroptosis in diabetes-induced skin lesions, we determined the indices of ferroptosis. We found that the expression of the lipid peroxide product MDA in the exudates of patients with diabetic foot was higher than that in the wound exudates of patients without diabetes mellitus (Figure 1A). A remarkable increase in MDA expression was also observed in the epidermis of diabetic rats compared with that in the controls (Figure 1B). To further validate these results, we measured the lipid peroxide product 4HNE. The histological results showed that 4HNE expression was significantly higher in skin tissues from patients and rats with diabetes mellitus than that from healthy subjects (Figure 1C). In line with prior research, an increase in ROS levels was observed in the skin of diabetic mice, especially in the epidermis (Supplementary Figure S1B). Furthermore, we investigated the morphological characteristics of ferroptosis using TEM. According to the TEM results, distinctive mitochondrial morphological features changed in the keratinocytes of diabetic rats, including smaller mitochondria, decreased mitochondrial ridges, and mitochondrial outer membrane rupture (Figure 1D). We also examined the expression levels of key enzymes involved in ferroptosis, ACSL4, and GPX4. Data revealed an up-regulation in both mRNA and protein expression for ACSL4, an indispensable component of lipid peroxide accumulation and ferroptosis, which were prominently increased in diabetic rats compared with control subjects (Figure 1E–1F). Immunohistochemical results suggested that ACSL4 was mainly highly expressed in epidermal keratinocytes of patients and rats with diabetes mellitus (Figure 1G). However, the expression of GPX4, the main anti-lipid peroxidation enzyme in ferroptosis, showed no significant difference among the groups (Supplementary Figure S1C). Collectively, the findings imply that ferroptosis contributes to the development of diabetic skin lesions, and the increased expression of ACSL4 may be a significant factor in this pathological process.

### Ferroptosis inhibitor promoted diabetic wound healing

**Figure 2:**
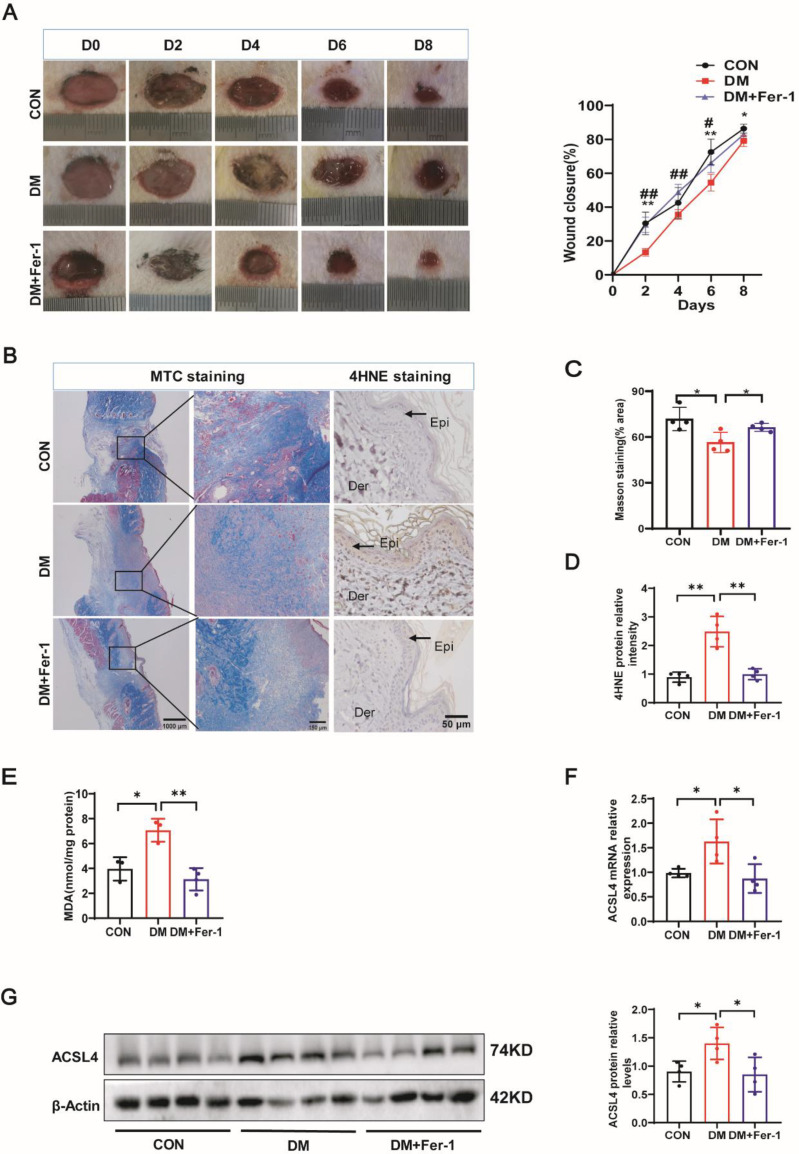
Ferroptosis inhibitor promoted diabetic wound healing. (**A**) Representative images of the wounds of control rats treated with control solvents and diabetic rats treated with Fer-1 or control solvents (*n* = 4). (**B and C**) Masson’s staining of the wounds of control and diabetic rats treated with the formulas, *n* = 4; original magnification 20 × 100. (**B and D**) Histological analysis of 4HNE levels in skin tissues from control and diabetic rats treated with the formulations (*n* = 4). Original magnification × 200. (**E**) MDA content in the wound tissues of control and diabetic rats treated with the formulations (*n* = 4). (**F and G**) Skin tissues from control and diabetic rats treated with the formulas were subjected to real-time PCR for ACSL4 (**F**) and immunoblotting (**G**), *n* = 4. The significance of CON versus DM is shown as * and that of DM versus DM+Fer-1 as **#**. Bars represent the mean ± SD; *^, **#**^ *P*<0.05; **^, **##**^ *P*<0.01; ***P<0.001.

To corroborate the presence of ferroptosis and its involvement in the diabetic wound repair process, diabetic rats received a subcutaneous injection of the ferroptosis-specific inhibitor Fer-1 and a negative PEG300 solvent on the edge of circular skin wounds once every other day for five cycles for 10 consecutive days. As shown in Figure 2A, the wound closure rate in diabetic rats administered with Fer-1 was notably quicker compared with the untreated diabetic control group. Fer-1 treated diabetic rats showed reduced wound width as observed by HE staining (Supplementary Figure S2A). Collagen is a wound-healing indicator. Figure 2B and C illustrate that MTC staining detected an increased density of newly formed collagen fibrils in diabetic rats administered with Fer-1 compared with those treated with the control solvent. Moreover, expression of 4HNE was the highest in the DM group but decreased significantly after intervention by Fer-1 (Figure 2B and D). Consistently, MDA expression in diabetic rats treated with Fer-1 was lower than the model group (Figure 2E). In addition, a reduction in ACSL4 mRNA levels due to Fer-1 treatment is shown in Figure 2F. Furthermore, Western blot results indicated a decline in ACSL4 protein expression post Fer-1 administration, as illustrated in Figure 2G. We validated this result using Immunohistochemistry (IHC) staining (Supplementary Figure S2B). Consistently, Fer-1 decreased the ferrous ions of the wound tissues of control and diabetic rats (Supplementary Figure S2C). The findings indicate that ferroptosis is a pivotal factor in the retarded healing process of diabetic wounds, and the administration of Fer-1 can counteract this effect, thereby enhancing wound repair.

### High glucose induced ferroptosis in keratinocytes

**Figure 3:**
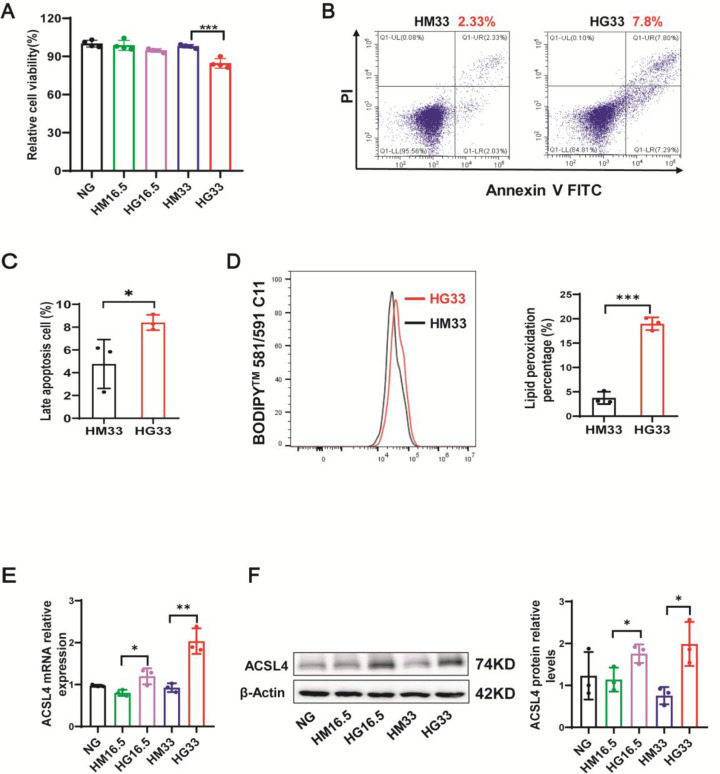
High glucose (HG) induced ferroptosis in keratinocytes. (**A**) Cell viability of human primary keratinocytes treated with NG (normal glucose: 5.6 mM of glucose), G (mid-high glucose: 16.5 mM of glucose (HG16.5); high glucose: 33 mM of glucose (HG33)), or M (16.5 mM: 5.6 mM of glucose + 10.9 mM of Mannitol (HM16.5); 33 mM: 5.6 mM of glucose + 27.4 mM of Mannitol (HM33) for 72 h, *n* = 3. (**B and C**) Rate of cell death in human primary keratinocytes treated with HM33 and HG33 for 72 h, *n* = 3. (**D**) Rate of lipid peroxidation in human primary keratinocytes treated with HM33 or HG33 for 72 h, *n* = 3. (**E**) ACSL4 mRNA levels in HaCaT cells treated with NG, HM16.5, HG16.5, HM33, and HG33 for 72 hours were measured by qPCR (*n* = 3). (**F**) ACSL4 protein levels in HaCaT cells treated with NG, HM16.5, HG16.5, HM33, and HG33 for 72 hours were measured by immunoblotting (*n* = 3). NG: normal glucose, HM16.5:5.6 mM of glucose + 10.9 mM of Mannitol, HG16.5:16.5 mM of glucose, HM33:5.6 mM of glucose + 27.4 mM of Mannitol, HG33:33 mM of glucose. Bars represent the mean ± SD; *P<0.05; **P<0.01; ***P<0.001.

To evaluate ferroptosis in keratinocytes under high-glucose (HG) stimulation, we conducted an investigation using human primary keratinocytes exposed to varying concentrations of mannitol and glucose for a duration of 72 h. Ferroptosis was assessed based on cell viability, cell death, lipid peroxidation, ferrous ions, and ACSL4 expression. We examined keratinocytes stimulated with different HG gradients and found that 33 mM HG can better model the phenotypes of diabetic skin. Thus, 33 mM HG was chosen for the *in vitro* experiments. It was noted that exposure to 33 mM HG levels led to a decrease in the viability of primary keratinocytes (Figure 3A) and increased cell death (Figure 3B and C). Lipid peroxidation accumulation is an important feature of ferroptosis, and we detected lipid peroxidation in 33 mM HG-stimulated keratinocytes. Ferrous ions probe showed that ferrous ions in HG-stimulated HaCaT cells were significantly higher (Supplementary Figure S3A). As shown in Figure 3D, 33 mM HG significantly increased the cellular lipid peroxidation levels. As ACSL4 is considered to be a key enzyme to trigger ferroptosis, we detected the ACSL4 expression in HG-stimulated keratinocytes. As expected, we observed that both mRNA and protein levels of ACSL4 were increased in primary keratinocytes following treatment with 33 mM HG (Supplementary Figure S3B,C). To further certify this result, the expression of ACSL4 mRNA and protein was measured in a human keratinocyte (HaCaT) cell line treated with 33 mM HG. Consistent with the results in human-derived primary keratinocytes, ACSL4 mRNA and protein levels were also increased in HaCaT cells treated with 33 mM HG (Figure 3E and F). The findings indicate that HG conditions might enhance the susceptibility of keratinocytes to ferroptosis, with ACSL4 potentially playing a role in this process.

### ACSL4 induced ferroptosis in keratinocytes

**Figure 4:**
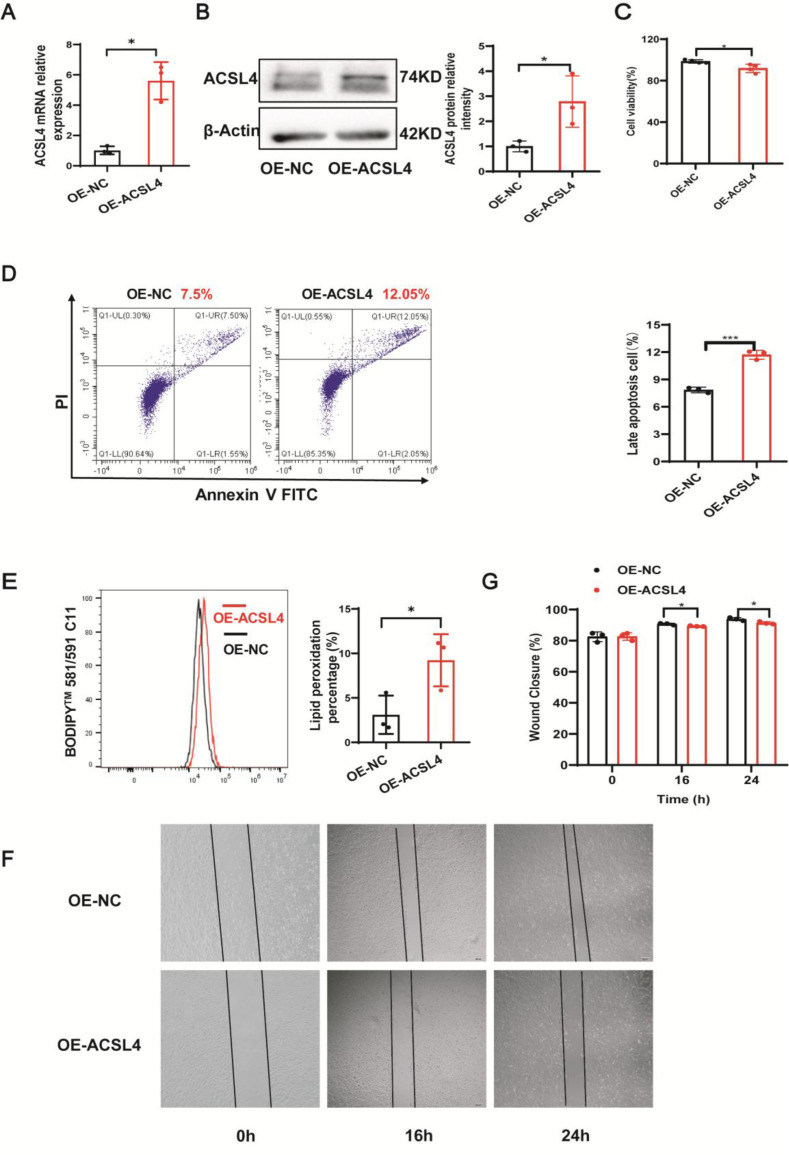
ACSL4 induced ferroptosis in keratinocytes. (**A**) qPCR analysis of ACSL4 mRNA expression in control and ACSL4 overexpression HaCaT cells, *n* = 3. (**B**) Immunoblotting analysis of ACSL4 protein expression in control and ACSL4 overexpression HaCaT cells. (**C**) Cell viability of HaCaT cells transfected with control and ACSL4 overexpression plasmids, *n* = 4. (**D**) Rate of cell death of HaCaT cells transfected with control and ACSL4 overexpression plasmids, *n* = 3. (**E**) Rate of lipid peroxidation of HaCaT cells transfected with control and ACSL4 overexpression plasmids, *n* = 3. (**F and G**) Representative images of *in vitro* wound-healing assays in HaCaT cells transfected with control and ACSL4 overexpression plasmids. HaCaT cells were scratched 72 hours after plasmids transfection, *n* = 3. Bars represent the mean ± SD; *P<0.05; **P<0.01. ACSL4, acyl-CoA synthetase long-chain family member 4.

Whether ACSL4 regulates ferroptosis-associated injury in keratinocytes *in vitro* remains unknown. To address this question, we have overexpressed (OE) ACSL4 in HaCaT cells. As shown in Figure 4A and B, the ACSL4 overexpression plasmid was successfully transfected. Consistent with our hypothesis, the results showed that after the overexpression of ACSL4, cell viability was decreased (Figure 4C) and cell death was significantly increased (Figure 4D). What is more, lipid peroxidation levels, an important feature of ferroptosis, were also increased after the overexpression of ACSL4 (Figure 4E). In addition, we investigated whether ACSL4 affected the migratory ability of HaCaT cells. The scratch assay outcomes indicated that an increase in ACSL4 expression led to reduced cell migration (Figure 4F and G). Overall, these findings imply that the up-regulation of ACSL4, a key controller of ferroptosis, influences cellular functions and could potentially result in high-glucose-induced ferroptosis within keratinocytes.

### N6-methyladenosine reader protein YTHDF2 interacted with ACSL4 mRNA through N6-methyladenosine-dependent way

**Figure 5 N6:**
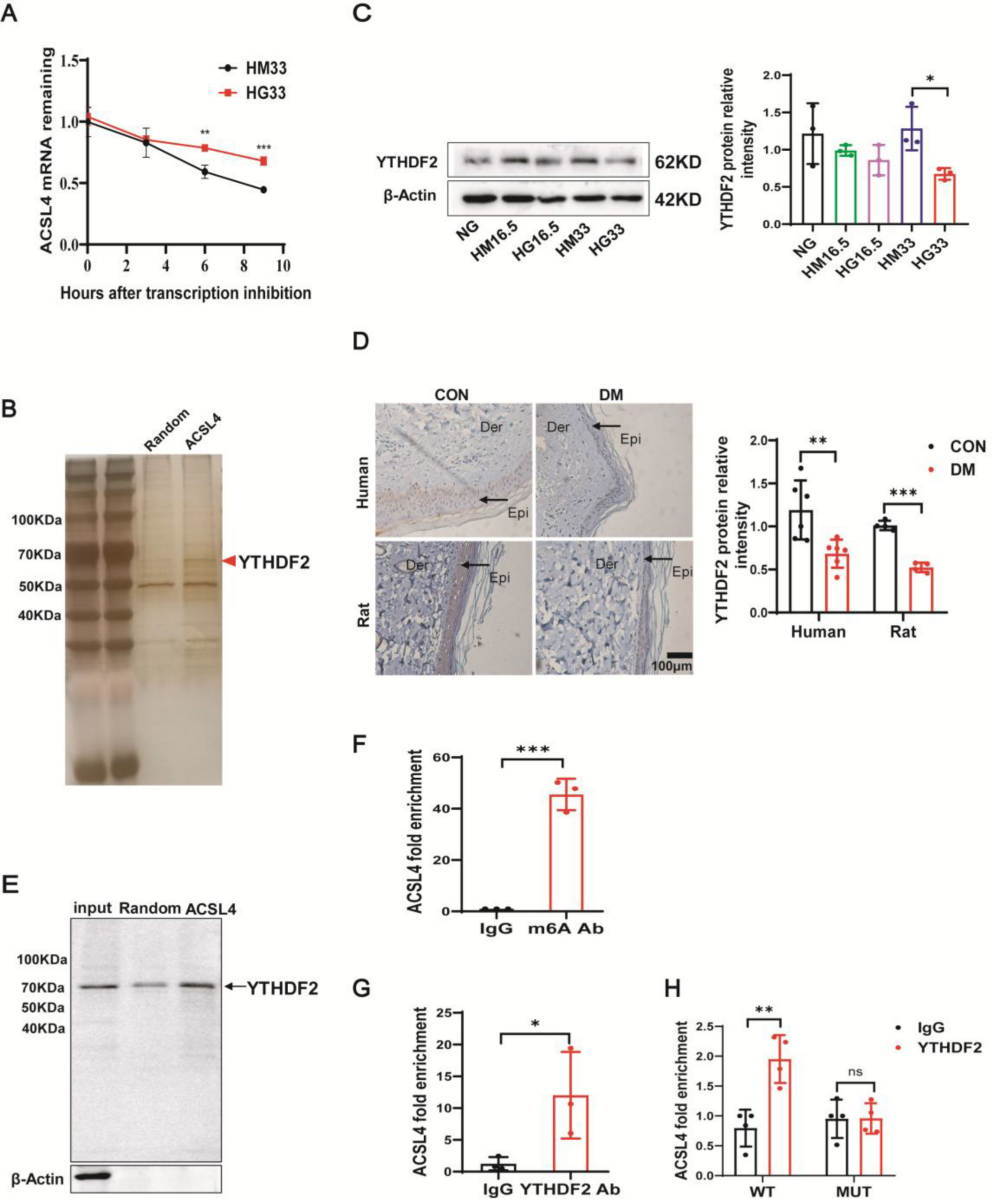
-methyladenosine reader protein YTHDF2 interacted with ACSL4 mRNA through N6-methyladenosine-dependent way. (**A**）High (33 mM) glucose and mannitol-stimulated HaCaT cells were treated with actinomycin D (5 μg/mL) for 0, 3, 6, and 9 hours. The expression of ACSL4 mRNA was analyzed using qPCR (*n* = 3). (**B**) Pull-down and silver staining analysis of ACSL4 mRNA-binding proteins in HaCaT cells. Arrowheads indicate YTHDF2 protein. (**C**) Immunoblot analysis of YTHDF2 protein expression in HaCaT cells treated with NG, HM16.5, HG16.5, HM33, or HG33 for 72 hours (*n* = 3). (**D**) Skin tissues from patients with diabetes (*n* = 3) and diabetic rats (*n* = 4) were subjected to histological analysis for YTHDF2 expression. Original magnification × 200. (**E**) Pull-down and Immunoblotting analyses of ACSL4 mRNA binding to YTHDF2 protein from HaCaT lysates. Arrowheads indicate YTHDF2 protein. (**F**) MeRIP-qPCR analysis of N6-methyladenosine levels in ACSL4 mRNA in HaCaT cells treated with normal glucose (*n* = 3). (**G**) RIP-qPCR and RNA affinity isolation analysis of the interaction between the YTHDF2 protein and ACSL4 mRNA in HaCaT cells (*n* = 3). (**H**) The N6-methyladenosine modification of ACSL4 WT plasmid and mutation (MUT) plasmid were transfected in the 293 T cells. RIP-qPCR and RNA affinity isolation analysis of the interaction between YTHDF2 protein and ACSL4 mRNA in 293 T cells. (*n* = 3). NG: normal glucose, HM16.5: 5.6 mM of glucose + 10.9 mM of mannitol, HG16.5: 16.5 mM of glucose, HM33: 5.6 mM of glucose + 27.4 mM of mannitol, HG33: 33 mM of glucose. Bars represent the mean ± SD; *P<0.05; **P<0.01; ***P<0.001. ACSL4, acyl-CoA synthetase long-chain family member 4; YTHDF2, YTH N6-methyladenosine RNA binding protein 2.

To further investigate the mechanism underlying increased ACSL4 expression in diabetic keratinocytes, actinomycin D was used to inhibit transcription in HaCaT cells treated with 33 mM mannitol and 33 mM HG. Interestingly, ACSL4 mRNA stability was enhanced in HaCaT cells under HG conditions (Figure 5A). Therefore, we speculated that ACSL4 mRNA is mainly regulated post-transcriptionally.

To explore the underlying mechanism, ACSL4 and random probes were used for pull-down assays to identify a protein that may regulate the ACSL4 mRNA stability. Mass spectrometry, combined with silver staining techniques, was employed to analyze proteins specifically bound to ACSL4 (Figure 5B). Surprisingly, we found that ACSL4 mRNA could bind with N6-methyladenosine modification proteins. N6-methyladenosine modification is an important factor in mRNA stability and splicing. Using the SRAMP website, possible N6-methyladenosine modification sites on the ACSL4 mRNA were identified (Supplementary Figure S4A). This suggests that the N6-methyladenosine modification protein may bind to the N6-methyladenosine modification sites of ACSL4 mRNA and regulate ACSL4 mRNA stability in an N6-methyladenosine-dependent manner. Then, we detected the expression of N6-methyladenosine modification proteins bound to ACSL4 mRNA in HG-stimulated HaCaT cells. The results showed that YTHDF2 was significantly and stably down-regulated in HG-stimulated HaCaT cells (Supplementary Figure S4B). Western blotting and IHC staining revealed that YTHDF2 protein expression was also decreased in diabetic keratinocytes (Figure 5C–5D). The results of Western blotting validation of YTHDF2 protein binding to ACSL4 mRNA were consistent with the mass spectrometry results (Figure 5E). MeRIP-qPCR was used to detect N6-methyladenosine modifications in ACSL4 mRNA. The results demonstrated that ACSL4 was enriched with the N6-methyladenosine antibody (Figure 5F). Using RIP-qPCR, we also found that ACSL4 was enriched with the YTHDF2 antibody (Figure 5G). Additionally, we mutated the N6-methyladenosine modification sites of ACSL4. Detailed information on the mutation of ACSL4 N6-methyladenosine sites was exhibited in Supplementary Data 1. RIP assay revealed that mutation of ACSL4 N6-methyladenosine sites diminished its interaction with YTHDF2 (Figure 5H). These results demonstrate that the N6-methyladenosine reader YTHDF2 may bind to ACSL4 mRNA in an N6-methyladenosine-dependent way.

### YTHDF2 regulated ACSL4 mRNA stability

**Figure 6:**
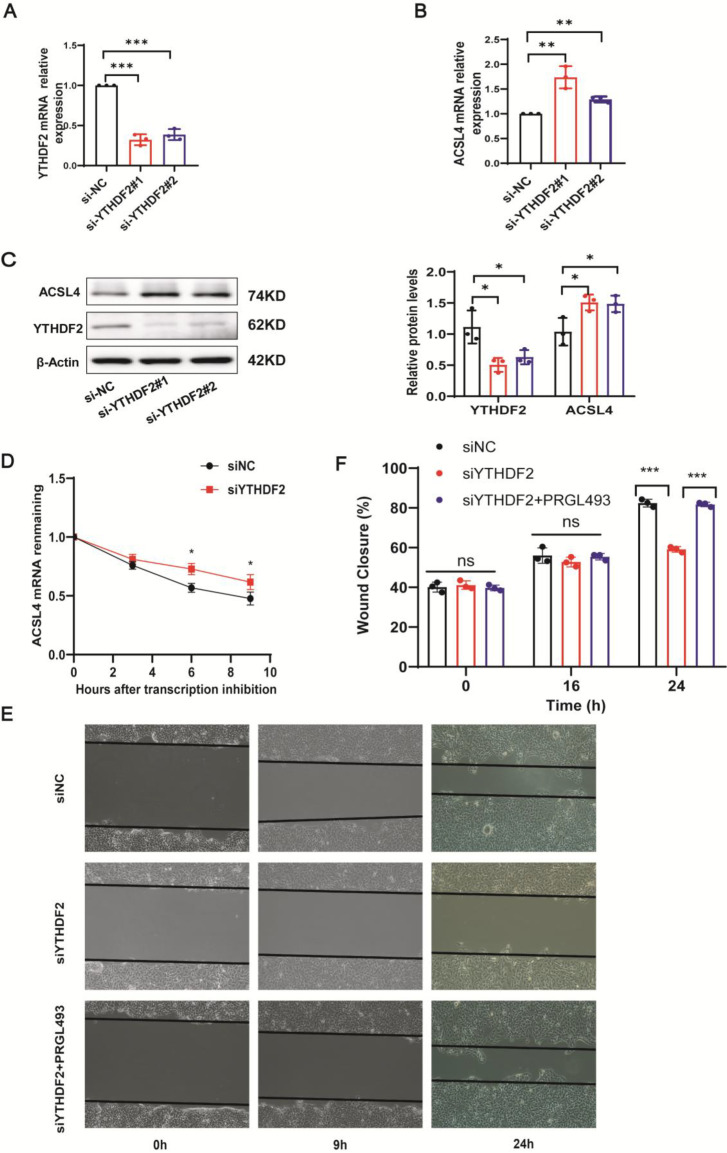
YTHDF2 regulated ACSL4 mRNA stability. (**A**) qPCR analysis of YTHDF2 mRNA expression in control and YTHDF2 knockdown HaCaT cells, *n* = 3. (**B**) qPCR analysis of ACSL4 mRNA expression in control and YTHDF2 knockdown HaCaT cells, *n* = 3. (**C**) Control and YTHDF2 knockdown HaCaT cells were subjected to immunoblotting of YTHDF2 and ACSL4 protein expression, *n* = 3. (**D**) Control and YTHDF2 knock down in HaCaT cells were treated with actinomycin D (5 μg/mL) for 0, 3, 6, and 9 hours, and cells were subjected to qPCR of ACSL4 mRNA. (**E, F**) Representative images of in vitro wound-healing assays in HaCaT cells transfected with control, siYTHDF2 and stimulated with ACSL4 inhibitor PRGL493. HaCaT cells were scratched at 48 hours after siRNAs transfection, *n* = 3. Bars represent the mean ± SD; *P<0.05; **P<0.01; ***P<0.001. ACSL4, acyl-CoA synthetase long-chain family member 4; YTHDF2, YTH N6-methyladenosine RNA binding protein 2.

To further verify that YTHDF2 regulates ACSL4 mRNA stability, we knocked down YTHDF2 in HaCaT cells using siRNA. The knockdown efficiency was validated using RT-qPCR (Figure 6A). YTHDF2 knockdown increased ACSL4 mRNA and protein levels (Figure 6B and C). To confirm the role of YTHDF2 as a negative ACSL4 regulator, we knocked down YTHDF2 in HaCaT cells and measured ACSL4 mRNA stability. We found that ACSL4 mRNA stability increased in YTHDF2-deficient cells (Figure 6D). To further verify YTHDF2 delays tissue repair through ACSL4, the ACSL4 inhibitor, PRGL493, was used to stimulate YTHDF2-knockdown HaCaT cells and a scratch test was performed. The result showed that PRGL493 can save the delayed wound closure resulting from siYTHDF2 (Figure 6E and F). Taken together, these findings suggest that YTHDF2 regulates ACSL4 expression and mediates ferroptosis in keratinocytes.

### Down-regulation of YTHDF2 resulted in ferroptosis in keratinocytes, leading to delayed wound healing in rats

**Figure 7:**
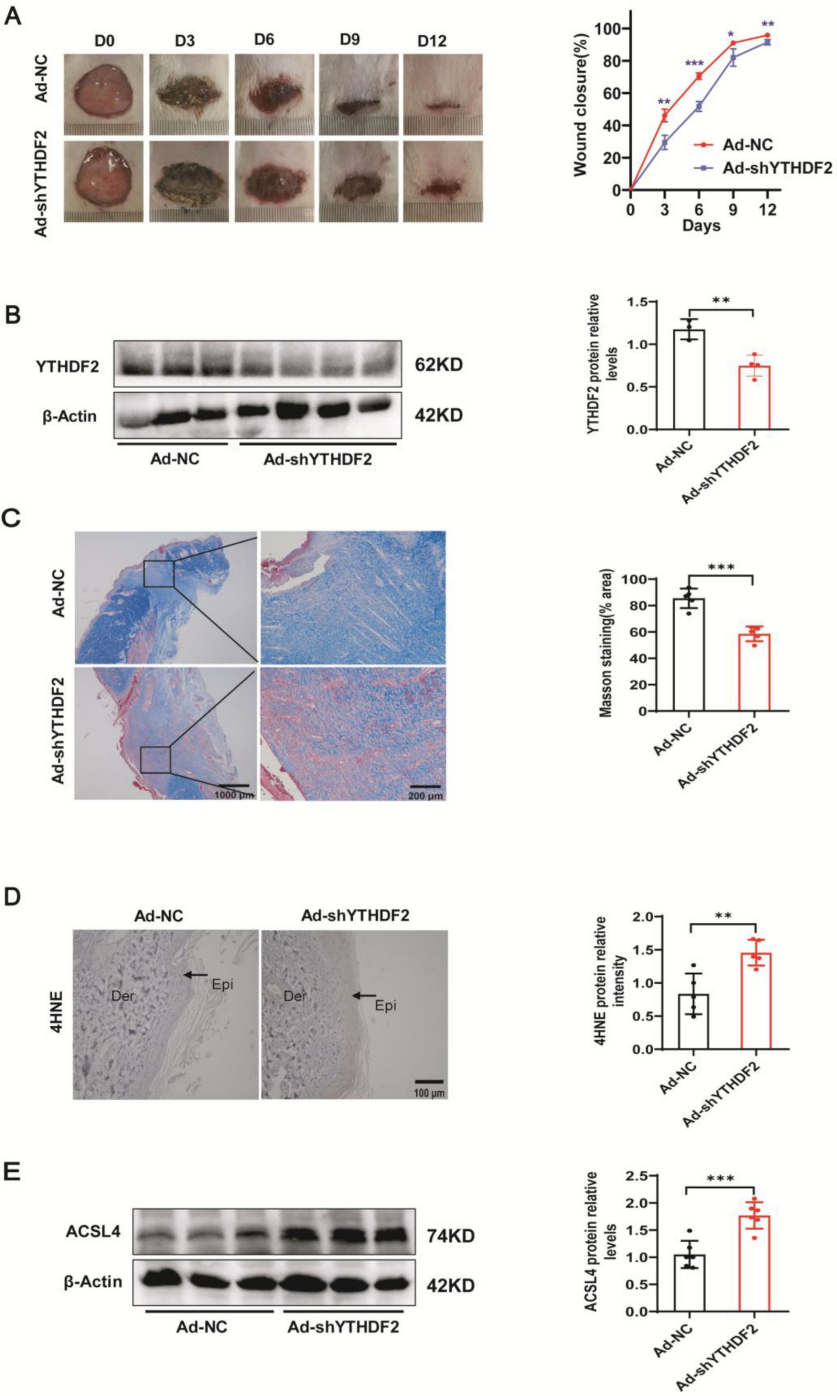
Down-regulation of YTHDF2 resulted in ferroptosis in keratinocytes, leading to delayed wound healing in rats. (**A**) Representative images of the wounds of SD rats treated with YTHDF2-knockdown adenovirus and control adenovirus (*n* = 5). (**B**) Skin tissues from SD rats treated with YTHDF2-knockdown adenovirus and control adenovirus were subjected to immunoblotting of YTHDF2, control adenovirus rats *n* = 3, YTHDF2-knockdown adenovirus *n* = 4. (**C**) MTC staining of the wounds of SD rats treated with YTHDF2-knockdown adenovirus and control adenovirus, *n* = 5. Original magnification × 20, × 100.(**D**) Histological analysis to detect 4HNE levels in skin tissues from SD rats treated with YTHDF2-knockdown adenovirus and control adenovirus, *n* = 5. (**E**) Skin tissues from SD rats treated with YTHDF2-knockdown adenovirus and control adenovirus were subjected to immunoblotting of ACSL4, *n* = 3. Bars represent the mean ± SD; *P<0.05; **P<0.01; ***P<0.001. SD, Sprague-Dawley; YTHDF2, YTH N6-methyladenosine RNA binding protein 2

To substantiate the functions of YTHDF2 and ACSL4 in the process of wound healing within a living organism, YTHDF2-knockdown and control adenoviruses were intradermally injected into the wound edges of WT SD rats. The ImageJ software was used to quantify the healed wound area at 0, 3, 6, 9, and 12 days after adenovirus injection. Notably, the rate of wound closure was markedly reduced in the YTHDF2 knockdown group compared with the control group (Figure 7A). The skin of the rats was collected 12 days after adenovirus injection. The Western blot technique confirmed successful reduction of YTHDF2 expression in the skin tissue (Figure 7B). IHC staining also confirmed successful knockdown (Supplementary Figure S5A). Consistent with the wound healing rate, SD rats treated with the YTHDF2-knockdown adenovirus exhibited wider wounds (Supplementary Figure S5B). Moreover, the density of the nascent collagen fibrils was lower in rats injected with YTHDF2-knockdown adenovirus than that in the control group (Figure 7C). An elevation in the concentration of the lipid peroxide product 4HNE was observed in the epidermis of rats with YTHDF2 knockdown (Figure 7D). More importantly, Western blot analysis revealed elevated levels of ACSL4, a marker for ferroptosis, in the skin from the YTHDF2 knockdown group relative to the control (Figure 7E). IHC staining revealed similar results (Supplementary Figure S5C). These results revealed that down-regulation of YTHDF2 protein in keratinocytes led to increased expression of the target ACSL4, resulting in ferroptosis and delayed wound healing.

## Discussion

**Figure 8:**
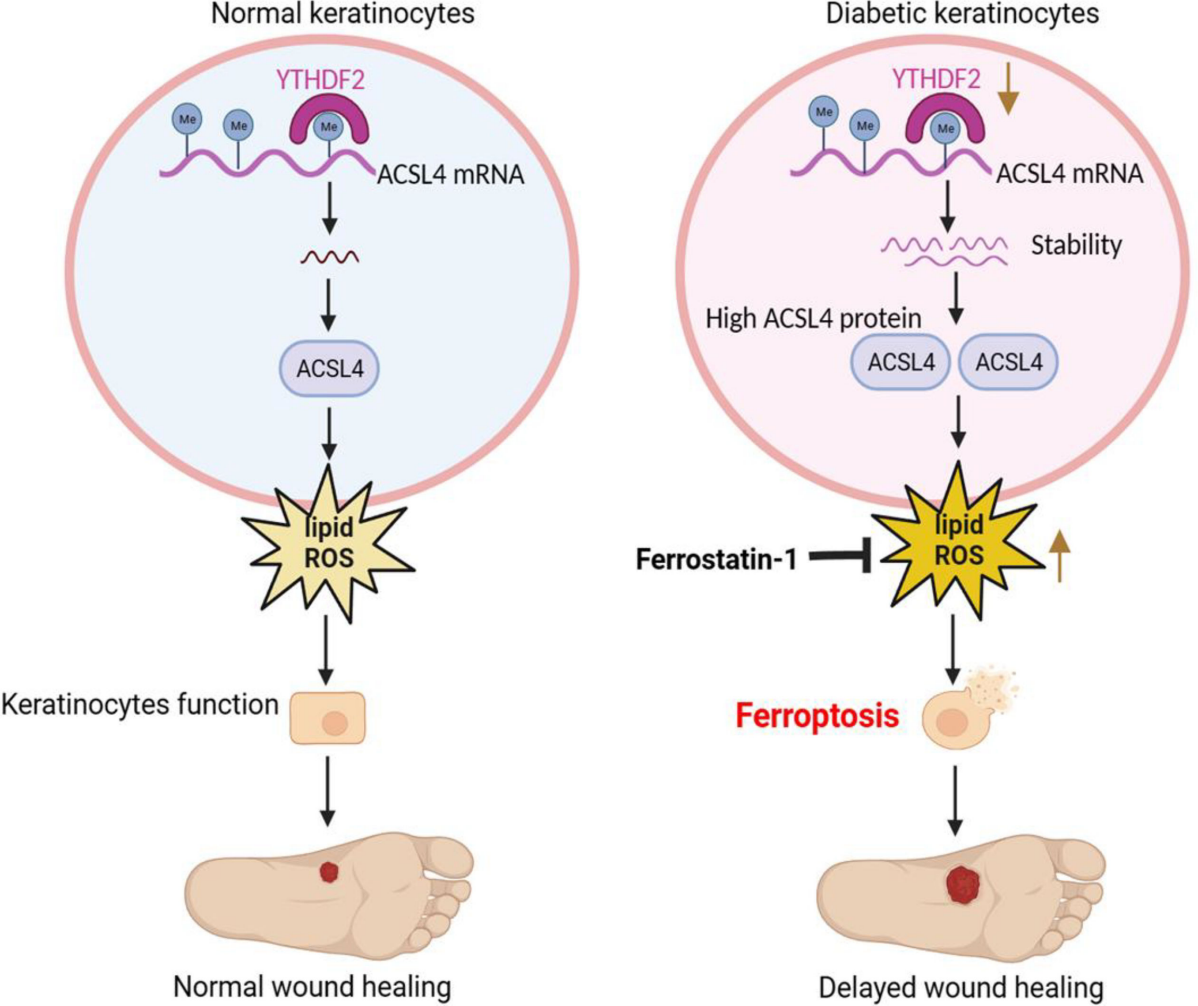
Schematic diagram of YTHDF2 regulating ACSL4-mediated ferroptosis of keratinocytes in diabetic wound healing. YTHDF2 interacts with and decays ACSL4 mRNA in an N6-methyladenosine-dependent manner in normal skin keratinocytes. In diabetic keratinocytes, YTHDF2 down-regulation induces ACSL4 mRNA up-regulation by decreasing ACSL4 mRNA decay, which causes lipid ROS in keratinocytes, ultimately leading to ferroptosis and delayed diabetic wound healing. ACSL4, acyl-CoA synthetase long-chain family member 4; YTHDF2, YTH N6-methyladenosine RNA binding protein 2.

Delayed skin wound remains a challenge for patients and clinicians. Programmed cell death, known as ferroptosis, which has recently been recognized, plays a substantial role in the development of various pathologies [37]. These include cancer, acute renal failure, neurodegenerative diseases, and metabolic conditions such as diabetes along with its complications [38,39]. This research, through the application of *in vitro* techniques and diabetic animal experiments, found that ACSL4 expression was elevated in diabetic keratinocytes, leading to the induction of ferroptosis and causing delayed diabetic wound healing. Mechanistically, we found that YTHDF2 regulated ACSL4 expression by regulating N6-methyladenosine-modified mRNA stability. Furthermore, YTHDF2 enhances ferroptosis via ACSL4 activation, thereby retarding wound repair processes in wild-type rats. Collectively, the results imply that the enhanced expression of ACSL4, induced by the reduced levels of YTHDF2 in the keratinocytes of diabetic skin, leads to ferroptosis and hampers the healing process of diabetic wounds (Figure 8). This research offers an innovative viewpoint on the healing of diabetic wounds, advancing the therapeutic strategies for such conditions.

The functional maintenance of keratinocytes in the skin during wound healing is critical [40], especially in diabetic wounds [41]. Diabetes is characterized by a chronic inflammatory state, which can affect iron metabolism homeostasis and cause iron overload in cells [42]. A significant increase in iron content has been observed in the renal tubules of patients with diabetic nephropathy [43]. ROS levels in tissues affected by HG also increased in the diabetic state. Interestingly, these pathological features of diabetes are key factors that trigger ferroptosis. This new type of cell death may serve as a therapeutic target for diabetic wound healing. Previous studies have found that the direct application of ferroptosis inhibitors to diabetic wounds can reduce the expression of markers such as oxidative stress and inflammation and promote diabetic wound healing [44]. However, another research found that the diabetic state can lead to the pathological accumulation of aging fibroblasts in diabetic wound tissue, which is mainly due to the decreased nuclear receptor coactivator 4 expression during ferritin autophagy. The accumulation of ferritin can reduce intracellular free iron ions, thus producing resistance to ferroptosis, while inducing ferroptosis using a ferroptosis inducer can significantly promote the process of wound repair in diabetic mouse models [45]. Therefore, the role of ferroptosis in wound healing in diabetes remains controversial. Our results showed that notable changes in ferroptosis, including excessive ROS, changes in the mitochondria, and increased ACSL4, occurred in diabetic skin, especially the epidermis, and in HG-stimulated keratinocytes. Based on these results, we believe that ferroptosis occurs in the skin of diabetic rats, particularly in the epidermis. Due to a lack of efficient therapy, diabetic wounds have become a leading cause of lower limb amputations worldwide [46]. To investigate a therapeutic approach and further verify the function of ferroptosis in the process of diabetic wound repair, ferroptosis inhibitors were directly applied to the wounds. These results showed that ferroptosis inhibitors promoted diabetic wound healing through ameliorating ferroptosis. Studies have found that diabetic conditions can trigger other types of cell death, including apoptosis, pyroptosis, etc. [47]. In our study, we did not exclude other types of cell death in diabetic skin but verified the importance of ferroptosis in diabetic wound healing using ferroptosis inhibitors. Our findings are consistent with a recent study showing that Fer-1 can accelerate diabetic wound healing via PI3K/AKT pathway activation [44]. These findings revealed a new mechanism for delayed diabetic wound healing and provided a new strategy for treating diabetic wounds.

The increase of ACSL4, a crucial ferroptosis determinant, can promote the development and progression of diabetic complications [48,49]. Nevertheless, the function of ACSL4 in the ferroptosis-induced death of keratinocytes has yet to be elucidated. Our results revealed that ACSL4 is not only an essential regulator that induces ferroptosis in diabetic keratinocytes but is also a key enzyme that leads to delayed diabetic wound healing. The findings of our study revealed a previously unknown mechanism where increased ACSL4 expression is crucial for high glucose-induced ferroptosis occurring in diabetic wound tissues or the skin, particularly in the context of keratinocyte mortality.

More importantly, we identified a new regulatory mechanism for ACSL4, in which its mRNA expression was regulated by YTHDF2 in an N6-methyladenosine-dependent way. In recent years, many types of post-transcriptional RNA modifications [28,50], including N6-methyladenosine, have demonstrated significant involvement in the control of RNA fate, such as RNA stability [51], RNA processing, and mRNA translation [52]. A previous study showed that N6-methyladenosine levels were down-regulated in HG-stimulated HaCaT cells [32]. In our study, we found that the N6-methyladenosine reader YTHDF2 could bind to the ACSL4 mRNA N6-methyladenosine site. As previously reported, YTHDF2 can promote mRNA decay through the recruitment of carbon catabolite repression-negative on the TATA-less deadenylase complex [53,54] and decreasing mRNA expression [34]. Consistent with previous studies, we found that YTHDF2 regulates the ACSL4 mRNA stability in keratinocytes. Given the important role of ACSL4 in triggering ferroptosis, there have been several reports on the mechanisms underlying the ACSL4 expression regulation. Studies found that ACSL4 transcription can be negatively regulated by many microRNAs, such as miR-211–5p, miR-204A-5p, miR-34A5p, miR-205, and miR-34a [55]. Moreover, cyclic adenosine monophosphate, SP1 transcription factor, tyrosine phosphatase SHP2, and proto-oncogene transcriptional coactivator YAP up-regulates the expression of ACSL4 [49,56]. Another study showed that arachidonic acid can induce ACSL4 protein degradation via the UBQ-proteasomal pathway without affecting ACSL4 mRNA stability in hepatic cells [57]. Currently, there are few studies on the regulation of ACSL4 mRNA expression by N6-methyladenosine RNA modification. A recent study reported that METTL14 up-regulation promotes pancreatic cell inflammation and ferroptosis by increasing ACSL4 expression in an N6-methyladenosine-dependent manner in severe acute pancreatitis [58]. This finding further confirms that ACSL4 mRNA is regulated by N6-methyladenosine RNA-methylated proteins. Our findings further enrich the mechanistic research on ACSL4.

Subsequent animal experiments further verified our results that down-regulation of YTHDF2 in the wounds of WT rats can increase the expression of ACSL4, modulate ferroptosis levels, and impair wound repair. Numerous studies have reported the involvement of N6-methyladenosine modification proteins, particularly YTHDF2, in modulating diabetes and its associated complications [59]. Up-regulation of YTHDF2 expression under diabetic conditions has been shown to suppress pathological neovascularization in the retinal tissues of diabetic mice [60]. Elevated YTHDF2 levels observed in the livers of diabetic mice promote ferroptosis in hepatocytes by inducing the degradation of GPX4 and SLC7A11, thereby exacerbating liver injury. Consequently, targeting YTHDF2 represents a potential therapeutic strategy for mitigating diabetes-induced liver damage [61]. Collectively, these studies suggest the potential of targeting YTHDF2 as a therapeutic strategy. At present, the clinical treatment of diabetic wound healing is mainly about blood glucose control [62], revascularization, wound repair, negative pressure wound treatment, stem cell transplantation [63], hyperbaric oxygen therapy [64], and so on. However, all these treatments to promote diabetic wound healing are still limited. Our *in vitro* and *in vivo* findings showed that targeting ferroptosis caused by YTHDF2-mediated ACSL4 elevation may be a new and effective strategy.

It must be acknowledged that our present study has some limitations. First, the sample size of clinical specimens used in our experiments was limited. In future studies, we will increase the sample size. Second, only male rats were used in our animal models, which may affect the generalizability of the conclusions. Furthermore, while we have demonstrated the impact of ferroptosis on diabetic wound healing, the potential influence of other programmed cell death modalities was not excluded. Future studies are needed to evaluate the effects of other cell death pathways on diabetic wound healing. The writers or erasers mediating altered N6-methyladenosine modification levels of ACSL4 mRNA under diabetic conditions remain unclear and require further investigation. Additionally, future studies are needed to explore the underlying causes of YTHDF2 down-regulation in the diabetic state. This will provide deeper insights into the precise regulatory mechanisms of the YTHDF2-ACSL4 axis and facilitate its clinical translation.

## Conclusions

In conclusion, we examined the role of ferroptosis in diabetic wound healing and delved into the associated mechanisms. Our findings were the first to demonstrate that ACSL4-induced ferroptosis in keratinocytes played a crucial role in delayed wound healing in hyperglycemic conditions. Furthermore, our findings indicated that YTHDF2, down-regulated in diabetic keratinocytes, can regulate the expression of N6-methyladenosine-modified ACSL4 mRNA and wound healing. This study also shed light on the novel concept that N6-methyladenosine modification may have a role in regulating ferroptosis. Collectively, our results offer novel perspectives on the mechanisms and potential therapeutic targets for diabetic wounds. The study also provides significant understanding of the pathogenic mechanisms of ferroptosis and potential treatment strategies for additional diabetic complications.

**Clinical Perspectives**

- ACSL4, a key enzyme of ferroptosis, leads to ferroptosis in keratinocytes in diabetic skin, making diabetic wounds difficult to heal.
- Low expression of YTHFD2 in keratinocytes in diabetic skin is an important reason for increased expression of ACSL4.
- Targeting YTHDF2-mediated ACSL4 elevation is expected to provide a new target for the treatment of diabetic wound healing.

## Supplementary Material

Online supplementary figure 1

Uncited online supplementary table 1

## Data Availability

Proteome resources can be found at PRIDE database (Accession number: PXD052755). Additional details and inquiries concerning the resources and reagents mentioned in this manuscript should be addressed to the primary contact Meng Ren(renmeng80@139.com).

## Open Access

The entire author team has reviewed and consented to the submission of the manuscript, and all members concur with its content being considered for publication in this journal.

## Abbreviations

ACSL4: Acyl-CoA synthetase long-chain family member 4
CCK8: Cell Counting Kit-8
DAB: diaminobenzidine
FBS: fetal bovine serum
Fer-1: ferrostatin-1
GPX4: glutathione peroxidase 4
HE: hematoxylin-eosin
HG: high glucose
4HNE: 4-hydroxynonenal
IHC: immunohistochemistry
KM: keratinocyte medium
MDA: malondialdehyde
MEM: minimum essential medium
MTC: Masson’s trichrome
OE: overexpressed
ROS: reactive oxygen species
RT-qPCR: quantitative real-time PCR
SD: Sprague-Dawley
STZ: streptozotocin
SiRNAs: small interfering RNAs
TNS: trypsin neutralization solution
WT: wildtype
YTHDF2: YTH N6-methyladenosine RNA binding protein 2
