# Supplementary material for: YTHDF2 regulates ACSL4-dependent ferroptosis of keratinocytes in diabetic wound healing
Source: Clin Sci (Lond). 2025 Aug 20;139(16):860–79. doi: 10.1042/CS20255877 (PMC12493160; doi:10.1042/CS20255877)
Supplement: Online supplementary figure 1 [file cs-139-16-CS20255877-s001.docx]

**Supplemental Figures**

**Supplementary figure 1. Elevated ACSL4 induces ferroptosis in diabetic skin**

(A) Representative HE staining of skin tissues from patients with diabetes (n=3) and diabetic rats (n=4), and the epidermal thicknesses of skin tissues were analyzed. Original magnification ×400.

(B) ROS was detected in the skin tissues of diabetic rats and controls.

(C) Skin tissues from diabetic patients (n=3) and diabetic rats were subjected to histological analysis of GPX4. Original magnification ×400.

Bars represent the mean ± SD; **p < 0.01; ns: no significant difference.


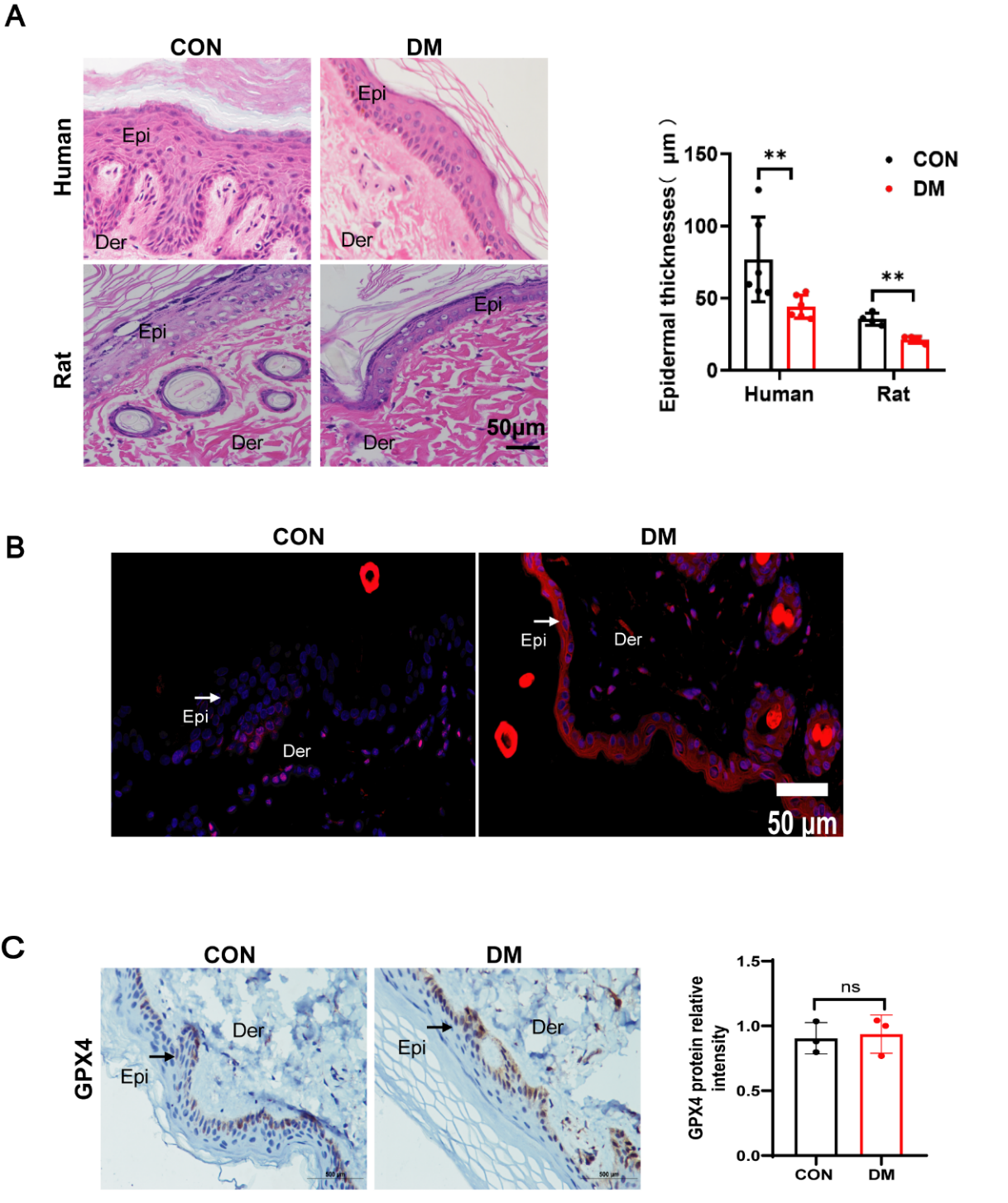


**Supplementary figure 2. Ferroptosis inhibitor promotes diabetic wound healing**

(A) HE staining of the wounds of control rats and diabetic rats treated with Fer-1 and control solvents, n=4.

(B) Skin tissues from control rats and diabetic rats treated with Fer-1 and control solvents subjected to histological analysis of ACSL4, n=4. Original magnification ×400.

(C) Ferrous ions of the wound tissues of control and diabetic rats treated with the formulations, n=3.

Bars represent the mean ± SD; *p < 0.05; **p < 0.01.

**
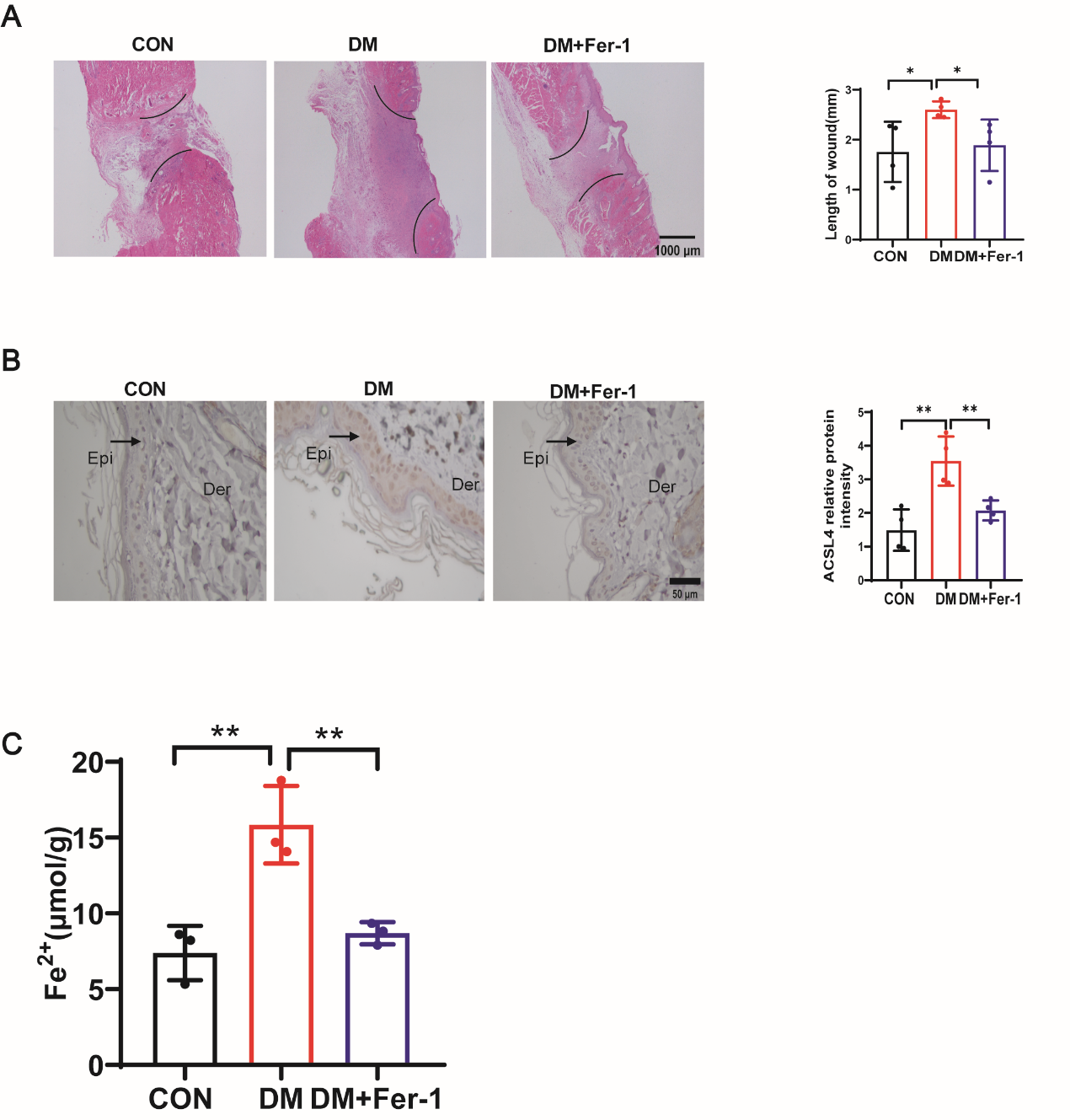
**

**Supplementary figure 3. High glucose（HG）induces ferroptosis in keratinocytes**

(A) Ferrous ions probe, FerroOrange, was used to detect the level of ferrous ions of HaCaT cells.

(B)ACSL4 mRNA of human primary keratinocytes treated with NG, HM16.5, HG16.5, HM33, and HG33 for 72 h were measured using qPCR, n=3.

(C) ACSL4 protein of human primary keratinocytes treated with NG, HM16.5, HG16.5, HM33, and HG33 for 72 h were measured by immunoblotting, n=3.

NG: normal glucose, HM16.5:5.6 mM of glucose + 10.9 mM of mannitol, HG16.5:16.5 mM of glucose, HM33:5.6 mM of glucose + 27.4 mM of mannitol, HG33:33 mM of glucose.

Bars represent the mean ± SD; *p < 0.05; **p < 0.01; ***p < 0.001.

**
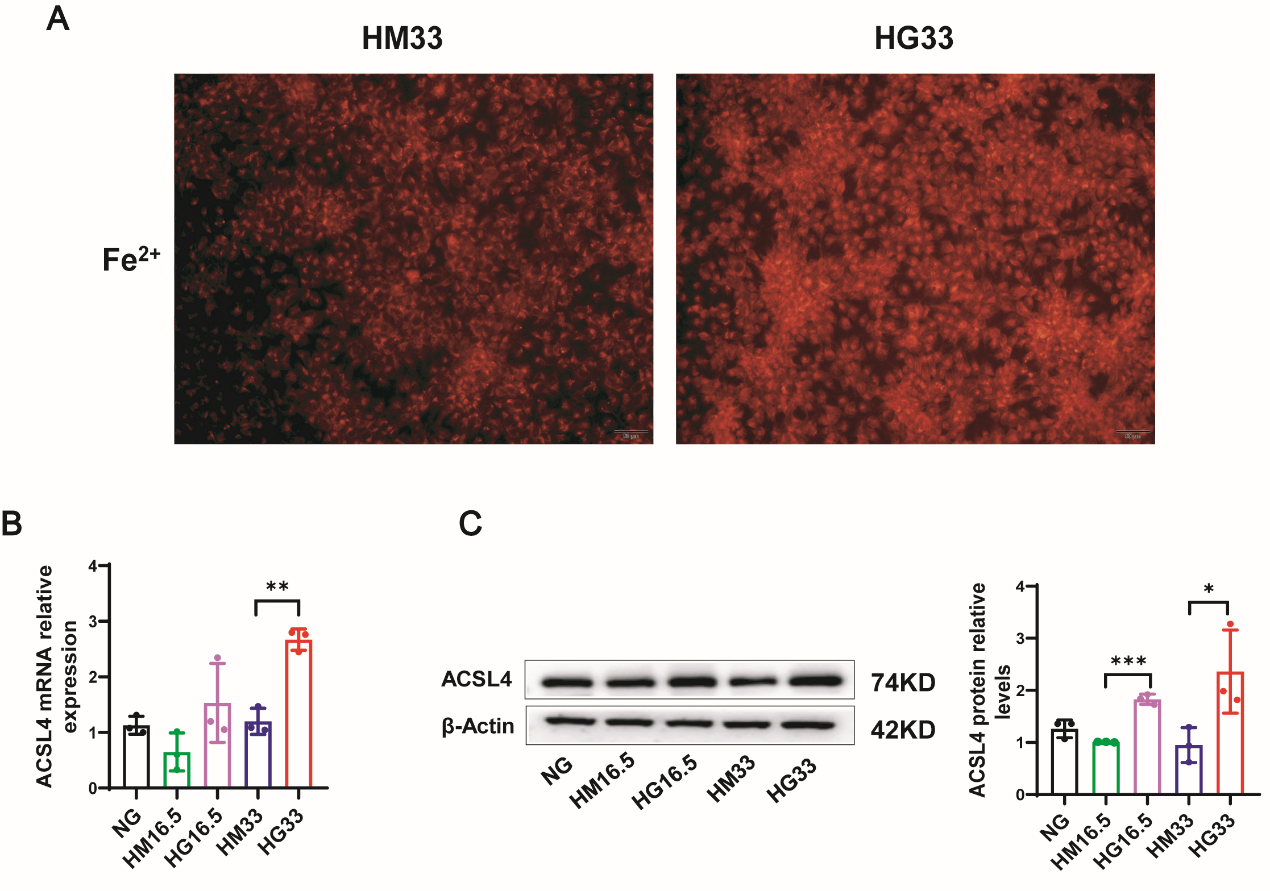
**

**Supplementary figure 4. m^6^A reader protein YTHDF2 binds ACSL4 mRNA through m^6^A-dependent way**

(A) SRAMP website was used to predict the m6A sites of ACSL4.

(B) RT-qPCR analysis of ACSL4 mRNA binding m6A modification proteins in the HaCaT cells treated with NG, HM16.5, HG16.5, HM33, and HG33 for 72 h, n=3.

NG: normal glucose, HM16.5:5.6 mM of glucose + 10.9 mM of mannitol, HG16.5:16.5 mM of glucose, HM33:5.6 mM of glucose + 27.4 mM of mannitol, HG33:33 mM of glucose.

Bars represent the mean ± SD; *p < 0.05.

**
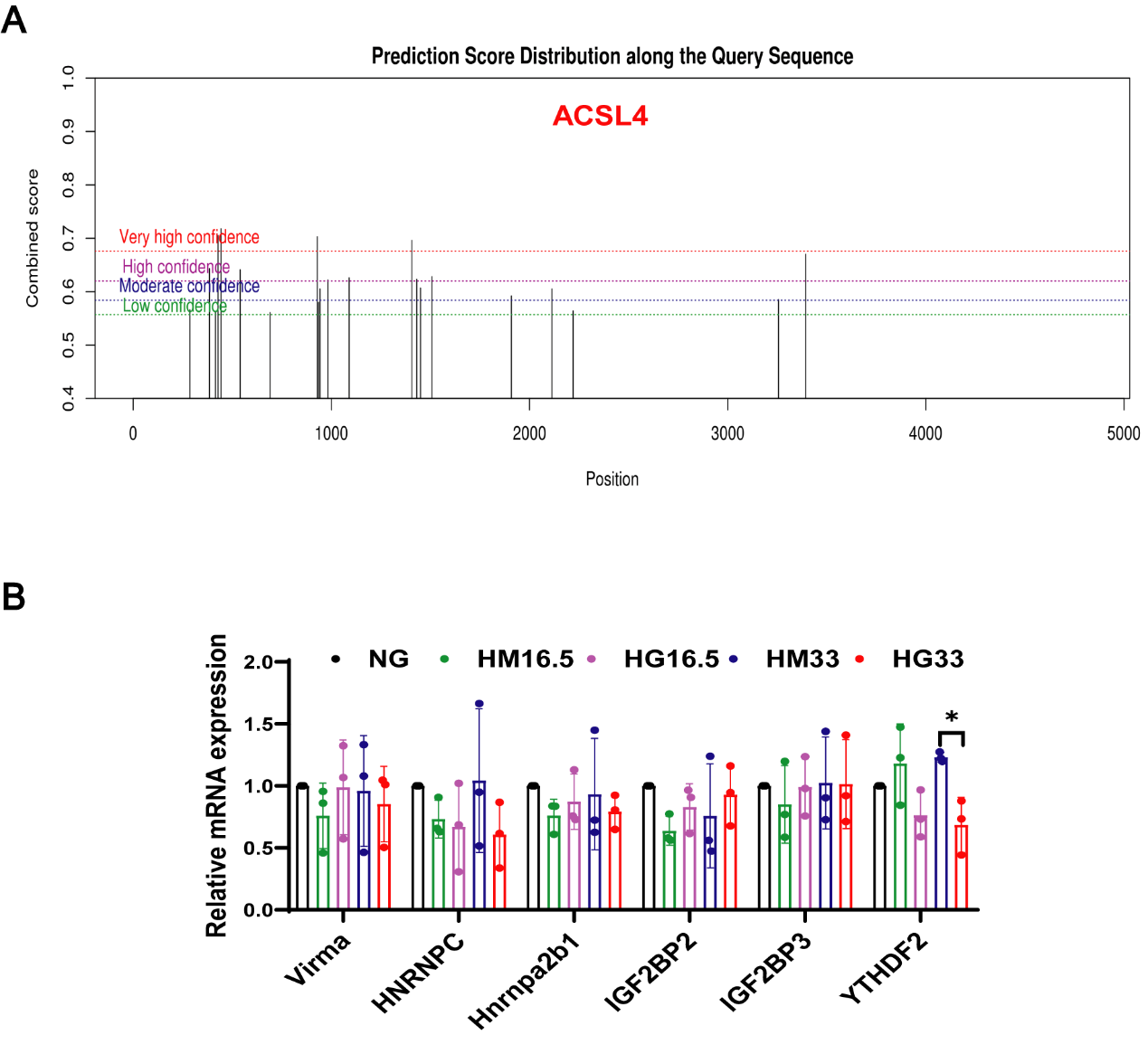
**

**Supplementary figure 5. YTHDF2 downregulation induces ferroptosis in the keratinocytes and delayed wound healing in rats**

(A) Skin tissues from SD rats treated with YTHDF2-knockdown adenovirus and control adenovirus were subjected to histological analysis of YTHDF2, n=5. Original magnification ×200.

(B) HE staining of the wounds of SD rats treated with YTHDF2-knockdown adenovirus and control adenovirus, n=5; Original magnification ×20

(C) Skin tissues from SD rats treated with YTHDF2-knockdown adenovirus and control adenovirus were subjected to Histological analysis of ACSL4, n=5. Original magnification ×200.

Bars represent the mean ± SD; *p < 0.05; **p < 0.01; ***p < 0.001.

**
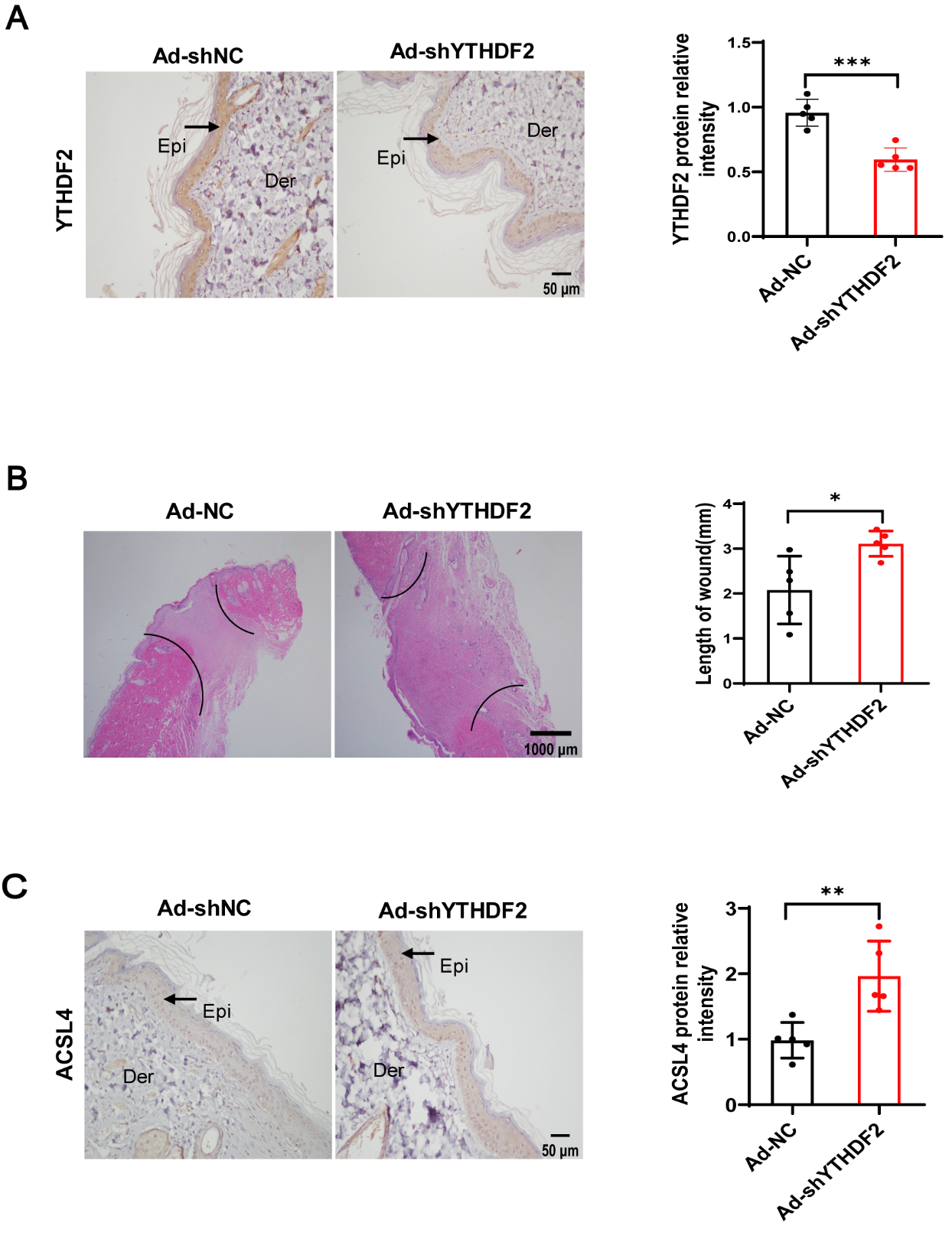
**
