# Supplementary material for: YTHDF2 regulates ACSL4-dependent ferroptosis of keratinocytes in diabetic wound healing
Source: Clin Sci (Lond). 2025 Aug 20;139(16):860–79. doi: 10.1042/CS20255877 (PMC12493160; doi:10.1042/CS20255877)
Supplement: Uncited online supplementary table 1 [file cs-139-16-CS20255877-s002.docx]

Supplementary table 1. siRNA sequences.

| siRNA Sequence |
| --- |
| si-h-YTHDF2-#1 GCACAGAAGTTGCAAGCAA  si-h-YTHDF2-#2 GGTAGCGGGTCCATTACTA |

h: human;

Supplementary table 2. Primer sequences.

| \| Gene \| Primer \| Sequence \| \| --- \| --- \| --- \| \| β-actin h \| Forward \| TGGAACGGTGAAGGTGACAG \| \| Reverse \| AACAACGCATCTCATATTTGGAA \| \| YTHDF2 h \| Forward \| AGCCCCACTTCCTACCAGATG \| \| Reverse \| TGAGAACTGTTATTTCCCCATGC \| \| ACSL4 h \| Forward \| ACTGGCCGACCTAAGGGAG \| \| Reverse \| GCCAAAGGCAAGTAGCCAATA \| \| β-actin r \| Forward \| GGAGATTACTGCCCTGGCTCCTA \| \| Reverse \| GACTCATCGTACTCCTGCTTGCTG \| \| YTHDF2 r \| Forward \| TCGAGCTGTTGTACGGTGAC \| \| Reverse \| GCGCCCTTCCTTAGGACTTT \| \| ACSL4 r \| Forward \| TCCATATCGCTCTGTCACGC \| \| Reverse \| GCTGTGAATCTCAAGCCCCT \| \| HNRNPA2B1 h \| Forward \| ATTGATGGGAGAGTAGTTGAGCC \| \| Reverse \| AATTCCGCCAACAAACAGCTT \| \| IGF2BP2 h \| Forward \| AGTGGAATTGCATGGGAAAATCA \| \| Reverse \| CAACGGCGGTTTCTGTGTC \| \| IGF2BP3 h \| Forward \| TATATCGGAAACCTCAGCGAGA \| \| Reverse \| GGACCGAGTGCTCAACTTCT \| \| HNRNPC h \| Forward \| GATGTACGGGTCAGTAACAGAAC \| \| Reverse \| AGCCCGAGCAATAGGAGGA \| \| VIRMA h \| Forward \| TGACCTTGCCTCACCAACTGCA \| \| Reverse \| AGCAACCTGGTGGTTTGGCTAG \| |
| --- | --- | --- | --- | --- | --- | --- | --- | --- | --- | --- | --- | --- | --- | --- | --- | --- | --- | --- | --- | --- | --- | --- | --- | --- | --- | --- | --- | --- | --- | --- | --- | --- | --- | --- | --- | --- | --- | --- | --- | --- | --- | --- | --- | --- | --- | --- | --- | --- | --- | --- | --- | --- | --- | --- | --- | --- | --- | --- |

h: human; r: rat

Supplementary table 3. Probes used in this study.

| Probes Sequence |
| --- |
| Random probe AAGTGTTCTTCGGACGCGAA  ACSL4 probe CAATTACGATTTCACCTCTG |
